# Carbamylation—A Pathologic Posttranslational Modification Affecting Platelet and Von Willebrand Factor Function during Uremic Kidney Disease

**DOI:** 10.1055/s-0044-1791550

**Published:** 2024-09-27

**Authors:** Rory R. Koenen

**Affiliations:** 1 Department of Biochemistry, CARIM School for Cardiovascular Diseases, Maastricht University, Maastricht, The Netherlands

Carbamylation is Instrumental in End-Stage Kidney Disease Coagulopathies: The Impact on von Willebrand Factor and Platelet Functionality

Chronic kidney disease imposes a heavy burden on the affected patients, due to the impaired clearance of metabolic waste products from the blood. This leads to increased concentrations of circulating so-called uremic toxins, a condition termed uremia. Uremia leads to numerous complications, contributing to the dramatically decreased life expectancy of patients with chronic uremic kidney disease. 1 One of such toxins found during uremia is carbamylic acid, which exists in an equilibrium that strongly favors urea. Elevated concentrations of urea however also lead to higher levels of carbamylic acid (and the carbamylate ion). Unlike the chemically inert urea, carbamylic acid modifies terminal amine moieties, such as found in lysine side chains of proteins. The result of this process, termed carbamylation, is a different terminal moiety accompanied by charge neutralization, potentially affecting the function of the modified protein (or of any amine-containing biomolecule).

Patients with end-stage kidney disease (ESKD) have an increased risk of hemostatic pathologies, 2 3 notably bleeding due to impaired platelet activity as a result of the action of uremic toxins and an impaired calcium homeostasis. 4 At the same time, there is also an increased risk of thrombotic events, which might be associated with acquired abnormalities in the coagulation system, or by activation of platelets during hemodialysis. 5 The pathophysiologic mechanisms that drive the tendency toward a bleeding or thrombotic phenotype during ESKD are still poorly understood. It has long been known that bleeding can be corrected by plasma cryoprecipitates or through administration of vasopressin, both augmenting plasma von Willebrand factor (VWF) concentrations. This suggests that VWF might be a major molecule affected by uremia and central in its bleeding pathology.

In the study by Babickova and Kałucka and colleagues, this current issue of *Thrombosis and Haemostasis* , the effects of carbamylation on platelet function were investigated with a focus on VWF. The authors commenced their investigations by treating isolated VWF with increasing concentrations of carbamylate and subsequently determining the modifications of the individual lysine residues by mass spectrometry. Interestingly, they identified lysine modifications in most VWF domains, notably A1 and A3 that are involved in platelet– and collagen–VWF interactions. Consequently, the interactions of VWF to collagen and platelets were shown to be impaired in solid-phase binding experiments implementing immobilized collagen or VWF, but only if the collagen or the platelets were also treated with carbamylate prior to the experiment. In fact, also the carbamylated platelets showed less binding to immobilized native untreated (healthy) VWF. Conversely, carbamylated VWF was found to bind more strongly to coagulation factor VIII, its natural binding partner in circulation. Interestingly, the binding of VWF to fibrinogen was not affected by carbamylation, indicating that carbamylation shows specific domain-dependent effects on VWF and its functions.

To characterize further functional consequences, the binding of carbamylated platelets on endothelial cells was investigated, revealing that carbamylation leads to a notable increase in platelet binding. To explore the underlying mechanisms that drive the altered platelet function after carbamylation, the release of thromboxane A2 and the presentation of phosphatidyl serine was measured after activation with thrombin or convulxin. Surprisingly, carbamylation inhibited the release of thromboxane A2 only after activation with thrombin, whereas no differences were found with convulxin. Similar stimulus-specific findings were obtained with regard to phosphatidyl serine exposure, albeit that thrombin stimulation led to an increase.

**Fig. 1:**
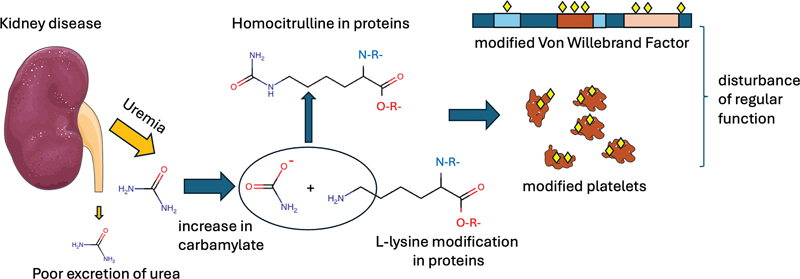
Pathologic protein carbamylation during uremia. Kidney dysfunction leads to a poor excretion of urea and other waste products. The accumulation of urea in the blood, a characteristic of uremia, leads to an increase in reactive carbamylate. Carbamylate can react with primary amines, e.g., converting lysine into homocitrulline, potentially leading to a loss of original function, as is exemplified by von Willebrand factor and platelets in the study by Babickova and Kałucka and colleagues.

This study by Babickova and Kałucka clearly demonstrates that carbamylation of VWF and of platelets leads to notable functional differences ( Fig. 1 ), and once more highlights that pathologic posttranslational modifications of biomolecules are an important aspect of ESKD to address. 6 However, it also leaves open some questions to be addressed in the future. First of all, it is unclear whether carbamylation of lysine residues in VWF leads to a loss of charge-dependent intermolecular interactions with its cognate binding site on platelets and on collagen, or whether the loss of binding is due to a loss of structural integrity and multimerization state of VWF. Second, it would be interesting to know whether carbamylic acid could enter the cells and modify intracellularly proteins, which would have implications for drug-induced VWF release (vasopressin). Third, the binding studies were performed under static conditions, whereas the binding of platelets to VWF and endothelial cells strongly depends on the flow regime. Finally, although the modification of platelets, endothelial cells, and VWF were performed under well-established conditions in vitro, it would be very interesting to extend the investigations to blood samples from uremic patients. This setup would also contribute to the question which of the many pathologic posttranslational modifications will contribute to a particular complication, leading to a much desired improvement in patient care and prognosis.
